# Superconductors in the PtPb_3_Bi Structure Type

**DOI:** 10.1021/acs.chemmater.6c01826

**Published:** 2026-08-19

**Authors:** Lior Verbitsky, Amira Merino, Scott B. Lee, Jaime M. Moya, Sigalit Aharon, Fatmagül Katmer, Sudipta Chatterjee, Grigorii Skorupskii, Josh Leeman, Gabrielle Carrel, Leslie M. Schoop

**Affiliations:** Department of Chemistry, 6740 Princeton University, Princeton, New Jersey 08544, United States

## Abstract

The quest for new superconductors is of both fundamental and technological importance. Recently, an artificial intelligence method correctly predicted PtPb_3_Bi to be a superconductor. In this work, we find superconductivity in the newly synthesized *M*Pb_4–*x*_Bi_*x*_ (M = Au, Pd, and Rh), of which PtPb_3_Bi is a member. When M = Ni, whose radius is considerably smaller, the structure instead collapses into the different Pb-substituted NiBi_3_ type. Interestingly, the stoichiometric parameter *x* shifts across the three compounds to keep the total valence-electron count close to 20 per formula unit. The superconducting transitions occur at 4.9, 4.2, and 3.4 K, for M = Au, Pd, and Rh, respectively. Using electrical resistivity, magnetization, and specific heat measurements, we establish the bulk nature of the superconducting state and determine the critical fields, characteristic length scales, and anisotropy ratios. All three compounds are moderately anisotropic conventional type-II superconductors, with modest upper critical field anisotropies of *H*_*c*2_^∥*c*^/*H*_*c*2_^⊥*c*^ ≈ 1.2 to 1.5. These results establish *M*Pb_4–*x*_Bi_*x*_ as a family of anisotropic superconductors and a platform for studying how site disorder and Pb–Bi mixing govern superconductivity in heavy-element intermetallics.

## Graphical abstract

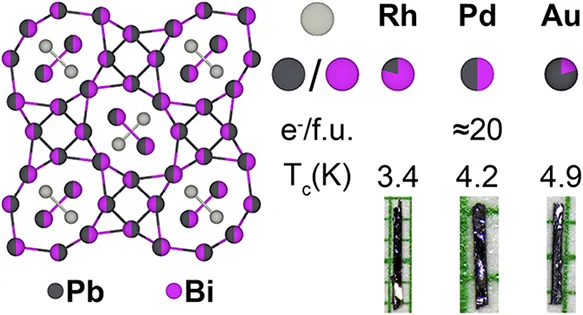

## Introduction

One of the intriguing features of superconductivity is that it often recurs within a given structure type, persisting robustly across substitutions and distortions. Prominent examples include the A15 compounds,^1^ the Chevrel phases,^2^ the cuprate high-*T*_*c*_ superconductors,^3,4^ and iron-based superconductors. The latter remain superconducting not only across a wide range of related structure types^5,6^ but also in structurally related, iron-free analogues.^7^ Identifying a new structure type therefore opens the door to systematic studies of structure–property relationships, in the spirit of the early empirical rules formulated by Matthias.^8^ Yet, even though many structural families have been discovered,^9^ together amounting to thousands of superconductors,^10^ predicting the superconducting transition temperature *T*_*c*_ of a given compound, let alone designing one with a higher *T*_*c*_, remains difficult and contested.

Because a first-principles calculation of *T*_*c*_ from the structure remains out of reach for most materials, recent work has turned to machine learning to identify structural descriptors that correlate with superconductivity. One such descriptor, the distribution of electron affinity differences between neighboring atoms, was recently shown to have predictive power for *T*_*c*_ and guided our discovery of superconductivity in PtPb_3_Bi.^11^ Until now, PtPb_3_Bi was the only known superconductor of its structure type (ICSD^12^ numbers 58834,^13^ 58835,[Bibr ref14] 197311 ^15^). The structure is tetragonal and is built from a Pb–Bi framework that encloses octagonal channels; within each channel, Pt–Pt and Pb-Pb dimers lying in the *ab* plane stack along the *c* axis in a mutually perpendicular fashion (Figure 1a).

**Figure 1:**
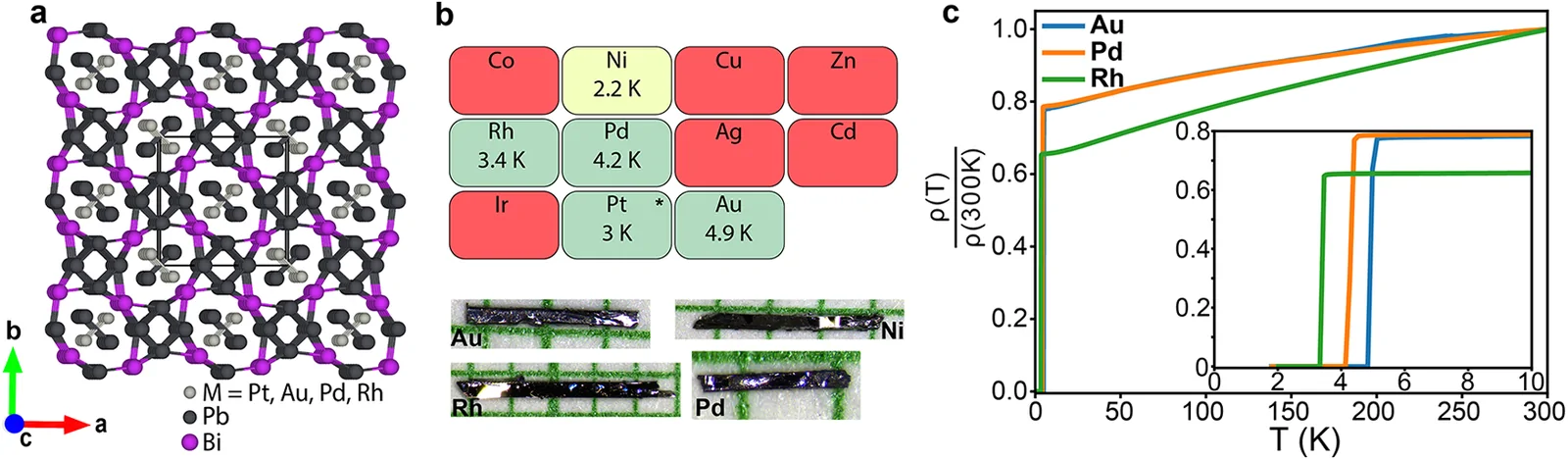
(a) Idealized crystal structure of the *M*Pb_4–*x*_Bi_*x*_ family, based on the archetypal PtPb_3_Bi structure. Gray, black, and purple spheres denote *M*, Pb, and Bi atoms, respectively, and the black rectangle outlines the unit cell. (b) Summary of the *M* substitution survey. Green cells mark successful synthesis of the *M*Pb_4–*x*_Bi_*x*_ structure together with the measured *T*_*c*_; the yellow cell marks the structurally related NiBi_3_ type; red cells mark attempts that did not yield a related structure. The asterisk on the Pt cell denotes the previously reported parent.^11,17^ Representative crystals on millimeter graph paper are shown below the grid. (c) Normalized resistivity for M = Au (blue), Pd (orange), and Rh (green) between 300 and 1.8 K. The inset highlights the sharp drop to zero resistivity at low temperature.

Based on the general idea of superconductivity “running in structure types”, and with, to the best of our knowledge, PtPb_3_Bi being the only structure of its type, we set out to expand the family by substituting other transition metals at the *M* site, while preserving the Pb–Bi framework. This straightforward strategy proved fruitful. We obtained three new superconductors, *M*Pb_4–*x*_Bi_*x*_ with M = Au, Pd, and Rh, all isostructural with PtPb_3_Bi. Because the measured Pb:Bi ratio deviates from the ideal 3:1 value found in PtPb_3_Bi, we denote these compounds *M*Pb_4–*x*_Bi_*x*_ throughout. When M = Ni, whose radius is considerably smaller, the octagonal channels collapse into hexagonal ones, and the material instead adopts the related NiBi_3_ structure type, which is itself a superconductor.^16^ Substitution with several other transition metals did not yield related compounds under the conditions explored. Taken together, these results establish *M*Pb_4–*x*_Bi_*x*_ as a small family of anisotropic, conventional type-II superconductors and a promising platform for studying how site disorder and Pb–Bi mixing influence superconducting properties.

## Experimental Section

### Crystal Growth

Single crystals of *M*Pb_4–*x*_Bi_*x*_ were grown by a Pb–Bi alloy self-flux reaction in a Canfield crucible set, using a 3:2 atomic ratio of Pb to Bi as the flux. The atomic fraction of *M* in the total charge was 11%, 5.5%, 2%, and 11% for Au, Pd, Rh, and Ni, respectively. The nominal atomic composition of the mixtures used was Au_11_Pb_53.4_Bi_35.6_, Pd_5.5_Pb_56.7_Bi_37.8_, Rh_2_Pb_58.8_Bi_39.2_, and Ni_11_Pb_53.4_Bi_35.6_. All elements were used as received: Pb shot (99.9%, Sigma-Aldrich), Bi pieces (99.999%, Sigma-Aldrich), Ni powder (99.9%, Thermo Scientific), Pd powder (99.95%, Thermo Scientific), Rh pieces (99.9%, Kurt J. Lesker), and Au powder (99.96+%, Thermo Scientific). The elements were weighed, mixed, and loaded into an alumina crucible, which was capped with a frit-disc and an inverted crucible. The Canfield set was sealed in a quartz tube that had been purged five times with argon and evacuated to below 50 mTorr, and the ampules were heated in programmable box furnaces. For Au and Pd, the charge was melted at 450 °C, held for 24 h, cooled to 200 °C at 5 °C/h and then to 150 °C at 2 °C/h, and held for 3 days before the still-hot ampule was inverted into a centrifuge and spun. For Rh, the mixture was heated to 1050 °C, soaked for 2 days, quickly cooled to 450 °C, cooled to 200 °C at 2 °C/h and then to 180 °C at 1 °C/h, and spun. For Ni, the charge was heated to 500 °C, held for 24 h, cooled to 350 °C at 1 °C/h, held for 3 days, and decanted. Before measurement, residual flux was removed by polishing and by etching in a mixture of glacial acetic acid and hydrogen peroxide.

### Structural and Chemical Characterization

The crystals were imaged using a scanning electron microscope (SEM), and initial elemental analysis was performed by energy-dispersive X-ray spectroscopy (EDS) on an FEI Quanta 200 FE-ESEM equipped with an Oxford Instruments X-Max EDX detector, and the data were processed with the Oxford Instruments AZtec software.

For precise determination, polished crystals were digested in a 9:1 mixture of trace-metal grade nitric acid (Fisher Chemical) and hydrochloric acid (Fisher Chemical) and diluted with Milli-Q water. Calibration curves of the measured elements were obtained by measuring a dilution series of a mixture of 1 g/L standards of gold, palladium, lead, bismuth, and rhodium (Sigma-Aldrich). The ratio of the elements in the resulting solutions was determined using an Agilent 5800 Inductively Coupled Plasma—Optical Emission Spectroscopy (ICP-OES) instrument. Single-crystal X-ray diffraction (SCXRD) was carried out at room temperature on either a Rigaku XtaLAB Synergy-S diffractometer (Mo *K*_α_ source, HyPix-Arc 150 hybrid-pixel detector) or a Bruker-AXS D8 Venture four-circle diffractometer (Mo *K*_α_ radiation, APEX2 CCD detector). Frame integration and data reduction were performed using CrysAlisPro for the Rigaku data and APEX2 for the Bruker data, and structures were solved and refined in Jana2020.^18^ SCXRD was also used to orient the crystals for the anisotropic measurements described below.

### Magnetization

Magnetic susceptibility was measured by vibrating sample magnetometry in a Quantum Design MPMS3 SQUID magnetometer. Crystals were mounted on quartz paddles with GE varnish, with the crystallographic *c* axis either parallel or perpendicular to the applied field. The dimensions *a*, *b*, and *c* of the cuboid crystals were measured digitally using a microscope for the calculation of the volume and the demagnetizing factors. The latter were estimated from the rectangular-cuboid expression 4*ab*/[4*ab* + 3*c*(*a* + *b*)].^19^ To minimize the effect of remnant fields in the superconducting magnet, each measurement was preceded by oscillating the magnet to zero field from 2 T at 15 K, well above the *T*_*c*_ of the samples.

### Electrical Transport

The resistivity was measured in a Quantum Design 9 T PPMS using the ETO option. Crystals were fixed to sapphire substrates with GE varnish and contacted in a four-probe geometry along the *c*-axis using four gold wires and silver paint. The mounted samples were placed on either a horizontal rotator or an electrical transport option puck, and the gold wires were soldered to the contact pads. A 1 mA drive current was applied at frequencies between 3 and 11 Hz.

### Heat Capacity

Heat capacity was measured with the heat-capacity option of the PPMS. Grease addenda (Apiezon N) were measured in zero field and in a high field between 1.8 and 300 K, after which polished crystals of known mass were placed on the premeasured grease.

### DFT

Density Functional Theory (DFT) calculations were performed on the experimental crystal structures with Vienna ab initio simulation package (VASP) 6.4.2.^20–23^ The Perdew–Burke–Ernzerhof (PBE) generalized gradient approximation (GGA)^24^ functional was used for exchange-correlation with the recommended potpaw-PBE.64 projector-augmented wave (PAW) pseudopotentials provided by VASP.^25,26^ The calculations used a converged (criterion of <10^–6^ eV) plane-wave energy cutoff of 550 eV Γ-centered 11 × 11 × 11 Monkhorst–Pack **k**-point grid^27^ and spin–orbit coupling. Band structures and densities of states were generated with the sumo package.^28^

## Results and Discussion

### Synthesis, Phase Formation, and Structure

Single crystals of *M*Pb_4–*x*_Bi_*x*_ (M = Au, Pd, Rh) adopting the PtPb_3_Bi structure type were obtained from a Pb–Bi self-flux. The Pb–Bi system is well suited for this purpose because it forms a deep eutectic near 126 °C.^29^ Since most solid-state syntheses are carried out at much higher temperatures, this unusually low-melting flux, perhaps together with the cost of the late transition metals involved, may be one reason these phases were previously overlooked.

The successful formation of each compound was first confirmed by SEM-EDS imaging of as-synthesized crystals (Figures S1–S3), where the *M*Pb_4–*x*_Bi_*x*_ crystals were observed, covered with drops of remnant flux. In order to obtain more precise elemental ratios, ICP-OES measurements were performed on digested solutions of polished crystals. The resulting empirical formulas are AuPb_2.78_Bi_1.35_, PdPb_2.08_Bi_1.92_, and RhPb_1.56_Bi_2.68_. The formulas clearly deviate from the perfect *M*Pb_3_Bi formula. Interestingly, based on the empirical ratios, the total number of valence electrons per formula unit is 20.6, 19.9, and 20.2 for M = Au, Pd, and Rh, or approximately 20 for all of the compounds, with AuPb_4–*x*_Bi_*x*_ being an outlier. Interestingly, additional randomly picked crystals of M = Au and Rh showed slightly different stoichiometries, AuPb_2.78_Bi_1.36_ and RhPb_1.50_Bi_2.78_, which result in electron counts per formula unit of 20.7 and 20.3. Thus, while each individual crystal might show slight variation in the exact stoichiometry, it appears that the general electron count remains similar. It is also possible that synthetic parameters could control the exact, or the range of, possible stoichiometries. The framework appears to keep the electron count per formula unit nearly constant: a change in the valence-electron count of M is offset by a compensating change in the Pb:Bi ratio (e.g., Pd → Au is balanced by Bi → Pb). Notably, the number of electrons per formula unit expected in stoichiometric PtPb_3_Bi is 19. While early reports lack experimental elemental analysis,^13^ the EDS results in a recent report on single-crystalline PtPb_3_Bi^11^ suggest an empirical stoichiometry that would result in an electron count between ≈ 19.7 (single point on the crystal) and ≈ 20.6 (for the entire crystal), thus approximately 20 as well. As a result, the *M*Pb_4–*x*_Bi_*x*_ phases are expected to have a homogeneity range, rather than just correspond to a line in the phase diagram, as reported for the PtPb_3_Bi structure.^13^ This suggests that a range of stoichiometries is possible for any given *M*, while conserving the total electron count of 20, that would correspond to a different density of *M* vacancies and a corresponding Pb/Bi ratio. Finally, the larger deviation of the total electron count in AuPb_4–*x*_Bi_*x*_ could either be an experimental artifact or potentially a manifestation of the aurophilic effect. Generally, the *M* atoms are in a capped distorted square antiprismatic environment, with the square antiprism consisting of Pb/Bi atoms, and the cap being another *M* atom in the same environment, effectively creating a dimer. In the refined crystal structures, the Au–Au dimer distance is 2.79 Å, well within the aurophilic range.^30^ It is thus potentially capable of hosting excess electron density, similar to previous reports of stabilization of 19-electron ternary phases.^31^ For M = Pd and Rh, the *M*-*M* distances are 2.84 Å and 2.85 Å, respectively—only slightly further apart than for Au. However, strikingly, the distance between the closest, quasi-dimarized Pb/Bi atoms in the rectangular base “sandwiched” between the two *M* atoms grows from 3.26 Å and 3.18 Å in PdPb_*4–x*_Bi*_x_* and RhPb*_4–x_* Bi*_x_* , respectively, to 3.50 Å in AuPb_*4-x*_Bi_*x*_. This further suggests a bonding interaction that is unusually strong between the two Au atoms, compared to that observed in the case of the other transition metals, and thus potentially being capable of hosting excess electrons.

We used SCXRD to verify and solve the crystal structure. The combination of pronounced Pb–Bi mixing, evident from the elemental analysis, with a lattice made up almost exclusively of heavy atoms, made the refinements challenging and sensitive to absorption-correction errors. Nevertheless, we obtained solutions with *R*(*I* > 3σ) < 5% for all three *M*Pb_4–*x*_Bi_*x*_ structures (Table S1). Because Pb and Bi have nearly identical scattering factors, we could not cleanly resolve their site occupancies with a laboratory X-ray source. Accordingly, every Pb/Bi site was modeled as a mixed Pb/Bi position with the relative occupancy fixed to the ICP-derived ratio; refining the M-site occupancy gave no improvement, so it was held fully occupied. The full crystallographic details are presented in Tables S1–S3, and the corresponding CIF files are provided as Supporting Information.

The substitution survey is summarized in Figure 1b. Together with the parent Pt, the transition metals Au, Pd, and Rh stabilize the PtPb_3_Bi structure type, indicating that the framework tolerates a range of *M* radii and *d*-electron counts and accommodates changes in the latter by adjusting the Pb:Bi ratio. Under the conditions we explored, several other metals did not form the structure: M = Ag, Cd, and Ir yielded mainly Pb_0.7_Bi_0.3_, M = Cu and Zn yielded mostly crystals of the elemental metal, and Co, which is highly immiscible in both Pb^32^ and Bi,^33^ remained largely unreacted up to 1200 °C. We cannot rule out that these or other ternaries could be stabilized under different synthetic conditions.

The unsuccessful substitutions can be rationalized along chemical lines. For M = Ag, Cd, Cu, and Zn, similar to the case of Co, the metals form no stable binaries with either Pb or Bi, and no known Pb–Bi ternaries, at ambient pressure.^34–38^ The failure of the 3*d* metals most likely reflects an *M* radius that is too small, and, in the case of Ag, a covalent radius that is too large. The failure of Cd may instead be a manifestation of the 20-electron conservation rule, since no positive *x* satisfies the rule for CdPb_4–*x*_Bi_*x*_. Ir is the most surprising absence: from the perspective of chemical intuition, and given the existence of stable Ir binaries with both Pb and Bi, one would expect it to behave like the metals that do form the PtPb_3_Bi structure. Such a compound may well exist, but its synthesis could be kinetically hindered by the low solubility of Ir in both Pb and Bi; indeed, even preparing new binary Ir phases can require multistep routes and spectator species.^39^

A distinct structural outcome occurs for the smallest metal we tested. When M = Ni, the product is a Pb-substituted version of the known superconductor NiBi_3_, namely NiBi_3–*x*_Pb_*x*_ (Figure S4), rather than the *M*Pb_3_Bi type.^16^ The two structures are nonetheless related: as the *M* radius decreases from Pd to Ni, the metal can no longer support the octagonal cage by forming planar dimers, and the cage collapses into a hexagonal channel in which the Ni atoms form zigzag chains along the *c* axis. For the refinement of the crystal structure of NiBi_3–*x*_Pb_*x*_ (Table S1), we again fixed the Bi/Pb ratio to the value obtained from EDS (Figure S4). From magnetic susceptibility (Figure S7), we find *T*_*c*_ ≈ 2.2 K for NiBi_3–*x*_Pb_*x*_, lower than the reported *T*_*c*_ ≈ 4 K.

Under high pressure conditions, Co has been reported to form the NiBi_3_-like CoBi_3_ structure, which also superconducts with a reported *T*_*c*_ ≈ 0.5 K.^40,41^ In addition, other high-pressure binaries of Bi with both Co^42^ and Cu^43^ are known to exist. The resulting structures are usually described as chains of the transition metal segregated from pillars of the Bi atoms, reminiscent of the complete segregation of the elements at ambient conditions. As some of the aforementioned high-pressure phases are metastable under ambient pressure, and could be isolated and characterized, high-pressure synthesis may therefore provide a route to additional *M*Pb_4–*x*_Bi_*x*_ compounds. Taken together with the aforementioned NiBi_3_-like RhBi_3_, as well as the isotypic La_2_NiBi^44^ and La_2_NiSb,^45^ we believe that the boundaries of *M*–Pb-Bi and related phases can be expanded even further.

### Superconducting Properties

Representative longitudinal resistivities between room temperature and 1.8 K are shown in Figure 1c. All three compounds are poor metals, with residual resistivity ratios *RRR* ≈ 1.3 to 1.5, and each shows a sharp drop to zero resistivity between 3 and 5 K. No additional transition from residual Pb or Pb–Bi alloy is observed, which, together with the etching procedure, indicates that the signal originates in the bulk crystals. Of note, similar critical temperatures were observed for at least five randomly picked crystals of each *M*, suggesting robustness toward the exact stoichiometry of the crystal which, according to the ICP-OES results, seems to slightly vary.

The tetragonal, channel-based structure naturally led us to examine the anisotropy of the superconducting state. Figure 2 shows the upper critical field phase diagrams, constructed by tracking the temperature of a 50% drop in resistivity from the normal-state value as a function of field (Figure 2a), which defines *H*_*c*2_(*T*), for the two principal field orientations; the raw curves for all orientations are given in Figure S5. The Au compound shows the highest *T*_*c*_ and the largest critical fields (Figure 2b), followed by Pd (Figure 2c) and Rh (Figure 2d). For all three, *H*_*c*2_ lies well below the weak-coupling Pauli limit, μ_0_ *H*_*p*_ ≈ 1.84 *T*_*c*_ (about 90 kOe for *T*_*c*_ ≈ 5 K).^46^ This places the materials in the orbitally limited regime, so that spin-paramagnetic pair breaking can be neglected despite the strong spin–orbit coupling expected from the heavy constituents. The low *RRR* furthermore places the samples in the dirty limit. We therefore fit the data to the orbitally limited Werthamer-Helfand-Hohenberg (WHH) equation,^47^ without spin or spin–orbit terms

$$\ln\left(\frac{1}{t}\right)=\psi \left(\frac{1}{2}+\frac{\mathrm{h̅}}{2t}\right)-\psi \left(\frac{1}{2}\right)$$

where ψ is the digamma function, *t* = *T*/*T*_*c*_, and *h̅* = 4*H*_*c*2_/[π^2^ *T*_*c*_ |d*H*_*c*2_/d*T*|_*T*_*c*__].

**Figure 2:**
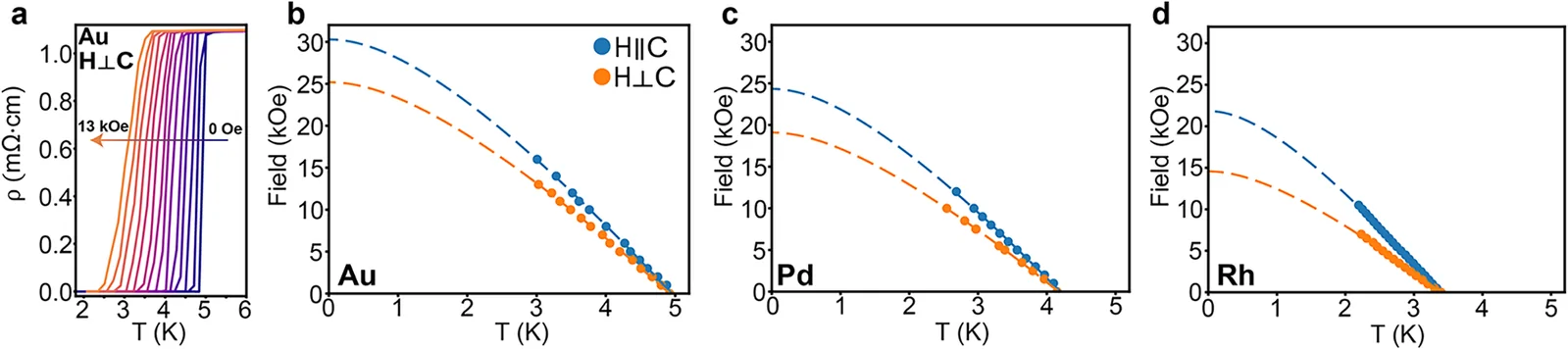
(a) Temperature-dependent resistivity of AuPb_4–*x*_Bi_*x*_ under increasing magnetic field at 1 kOe increments perpendicular to the *c* axis, shown as a representative example. (b–d) Upper critical field phase diagrams obtained by fitting the experimental points (dots) to the WHH model (dashed lines) for M = Au, Pd, and Rh, for fields parallel (blue) and perpendicular (orange) to the *c* axis.

Extrapolating to zero temperature gives *H*_*c*2_^∥*c*^(0) = 30, 24, and 22 kOe for Au, Pd, and Rh, respectively. Within the anisotropic Ginzburg–Landau (GL) theory,^48^ the corresponding *H*_*c*2_^⊥*c*^ values yield modest anisotropy ratios γ_ξ_(0) = *H*_*c*2_^∥*c*^(0)/*H*_*c*2_^⊥*c*^(0) = 1.2, 1.3, and 1.5 for Au, Pd, and Rh. A longer coherence length along *c* is consistent with the quasi-one-dimensional character of the structure. The GL coherence lengths follow from *H*_*c*2_^∥*c*^ = Φ_0_/(2πξ_*ab*_^2^) and *H*_*c*2_^⊥*c*^ = Φ_0_/(2πξ_*ab*_ξ_*c*_), with Φ_0_ the flux quantum, and are collected in Table 1 along with the other quantities discussed below.

**Table 1:** Superconducting Parameters of the *M*Pb_4–*x*_Bi_*x*_ Compounds for Fields Parallel and Perpendicular to the *c* Axis^a^

Element	Orientation	*T*_*c*_ (K)	*H*_*c*2_ (kOe)	ξ (nm)	γ_ξ_	*H*_*c*1_ (Oe)	λ (nm)	γ_λ_^–1^	κ
Au	∥*c*	4.9	30	12.5	1.2	53	222	1.6	34
	⊥*c*		25	10.0		77	355		24
Pd	∥*c*	4.2	24	14.8	1.3	45	196	1.9	33
	⊥*c*		19	11.6		78	383		21
Rh	∥*c*	3.4	22	18.4	1.5	28	224	2.2	40
	⊥*c*		15	12.3		54	491		22

a For ease of comparison with *γ*_ξ_, *γ*_*λ*_^–1^ is tabulated. For ξ, λ, and κ, ∥*c* and ⊥*c* refer to the *c* and *ab* axes, respectively.

To confirm that the superconductivity is a bulk property, we measured the magnetization across *T*_*c*_. Demagnetization-corrected zero-field-cooled (ZFC) and field-cooled (FC) susceptibilities under a 5 Oe field parallel to the *c* axis (Figure 3a–c) show strong diamagnetic shielding, with the ZFC signal approaching full flux expulsion, and no additional transition from Pb or Pb–Bi alloys. The ZFC demagnetization-corrected shielding fractions are found to be 4πχ_*v*_ ≈ −1.1 to −1.2, slightly exceeding the ideal full-shielding value. This modest overcorrection is attributed to uncertainty in the sample dimensions and the cuboidal approximation^19^ used for the demagnetization factor, and is consistent with full, bulk shielding within geometric uncertainty.

**Figure 3:**
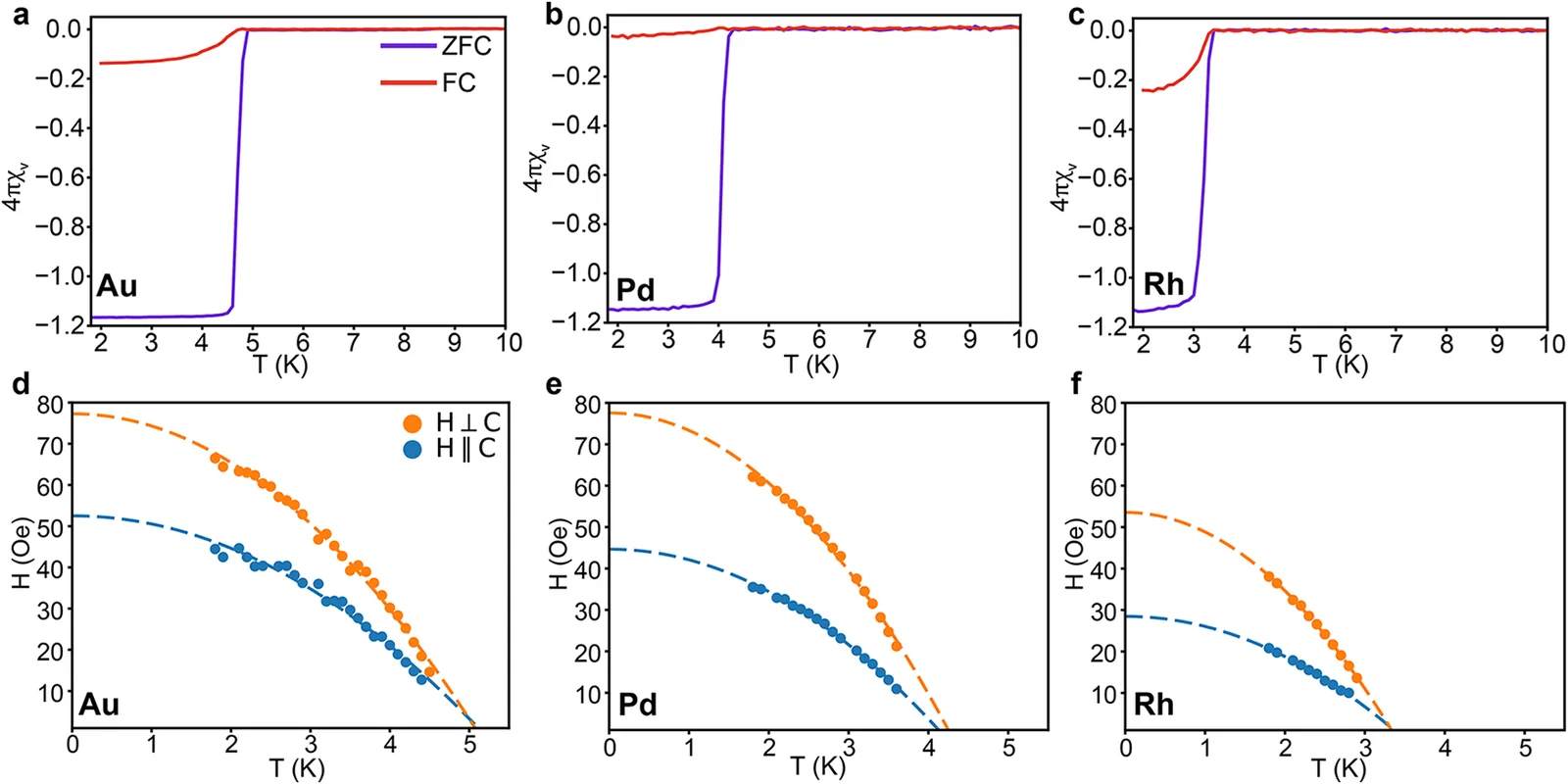
(a–c) Magnetic susceptibility under a 5 Oe bias field for M = Au, Pd, and Rh under zero-field-cooled (purple) and field-cooled (red) conditions. (d–f) Lower critical field phase diagrams, with *H*_*c*1_(*T*) extracted from the Meissner curves (dots) and fit to *H*_*c*1_(*T*) = *H*_*c*1_(0)­[1 – (*T*/*T*_*c*_)^2^] (dashed lines) for *H*⊥*c* (orange) and *H*∥*c* (blue).

We then determined the lower critical field *H*_*c*1_ from the deviation of the Meissner curves from linearity at several temperatures and both orientations (Figure S6). The resulting phase diagrams (Figure 3d–f) are fit to *H*_*c*1_(*T*) = *H*_*c*1_(0)­[1 – (*T*/*T*_*c*_)^2^] to extract *H*_*c*1_(0). The penetration depth along principal axis *i*, λ_*i*_, then follows from the tetragonal GL relations^49^ $H_{c1}^{∥c}=\frac{\Phi_{0}}{4\pi \lambda_{\mathrm{ab}}^{2}}\left(\ln\kappa_{c}+0.497\right)$ and $H_{c1}^{⊥c}=\frac{\Phi_{0}}{4\pi \lambda_{\mathrm{ab}}\lambda_{c}}\left(\ln\kappa_{\mathrm{ab}}+0.497\right)$ , with κ_*c*_ = λ_*ab*_/ξ_*ab*_ and $\kappa_{\mathrm{ab}}=\sqrt{\lambda_{\mathrm{ab}}\lambda_{c}/\left(\xi_{\mathrm{ab}}\xi_{c}\right)}$ . As expected from anisotropic GL theory, the penetration-depth anisotropy is the inverse of the coherence-length anisotropy, γ_λ_ = γ_ξ_^–1^, and Table 1 shows that the measured γ_λ_^–1^ values follow this trend. Finally, all of the GL parameters satisfy κ ≫ 1/√2, confirming that the compounds are type-II superconductors.

As a final and independent test of bulk superconductivity, we measured the specific heat across *T*_*c*_ in zero field and at 50 kOe, above the upper critical fields extrapolated for the compounds. The Bardeen-Cooper-Schrieffer (BCS) theory predicts a jump in the electronic specific heat of Δ*C*_*p*_^*e*^/(*γT*_*c*_) ≈ 1.43, where γ is the Sommerfeld coefficient. As fitting the zero-field, high-temperature data to *C*_*p*_/*T* = γ + β *T* ^2^ + δ *T* ^4^ consistently returned unphysical parameters, we used a previously reported method for the related NiBi_3_,^50^ estimating γ from 50 kOe measurements (well above *H*_*c*2_) between 1.8 and 2.3 K (Figure S8).

We obtained γ values of 12.8, 12.3, and 8.6 mJ mol^–1^ K^–2^ for Au, Pd, and Rh, values typical of intermetallics, and generally tracking with the observed *T*_*c*_. By fitting zero-field data just above the transition temperature to the same equation and subtracting it from the data points at the transition, we could get the change in the heat capacity Δ*C*_*p*_. By using the γ values previously obtained from the 50 kOe, low temperature data, we find Δ*C*_*p*_/(γ*T*_*c*_) = 1.50, 1.58, and 1.60 for Au, Pd, and Rh. These values, near or slightly above the BCS expectation, indicate bulk superconductivity with moderate coupling strength. Figure 4 illustrates the analysis for Au, where the high-field, low-temperature γ defines the baseline from which the jump Δ*C* is measured; the inset shows the high-temperature Debye fit and the full suppression of the transition by a 50 kOe field. The corresponding analyses for Pd and Rh appear in Figures S9 and S10. Note the uncertainty in the specifics of the heat capacity analysis due to artifacts seen in some samples (shown in Figures S9–S10). Nonetheless, the heat capacity confirms bulk superconductivity. Taken together with the shielding fractions from magnetization, the specific heat establishes the bulk nature of the superconducting state.

**Figure 4:**
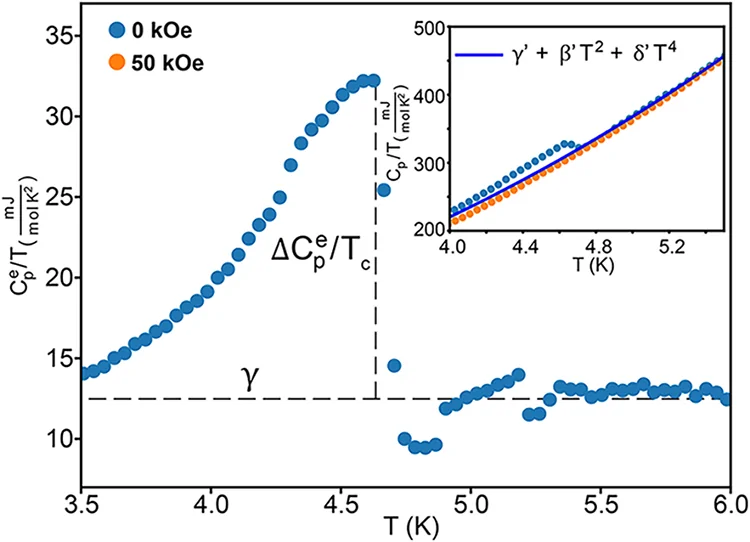
Electronic specific heat *C*_*p*_^*e*^/*T* of AuPb_4–*x*_Bi_*x*_ across the superconducting transition. Dashed lines mark the Sommerfeld coefficient γ, extracted from 50 kOe data at low temperature, and the jump Δ*C*_*p*_^*e*^/*T*_*c*_. The inset shows the raw total specific heat *C*_*p*_/*T* in zero and high field, with the high-temperature fit as the blue line.

### DFT Calculations

DFT calculations were performed in order to gain further insight into the structural and electronic properties of the compounds, as well as of PtPb_3_Bi (from ICSD^12^ 58834 ^13^). Since the actual Pb–Bi distribution is not known, for the sake of comparison, we assumed perfect *M*Pb_3_Bi structures, where each mixed Pb/Bi site was replaced by a fully occupied site of either Pb or Bi to mimic a stoichiometrically perfect PtPb_3_Bi. In addition, we used the experimental structures as solved and refined from the SCXRD measurements. As such, our calculations are limited to being qualitative in nature. As expected from the dimensionality and the bonding in the compounds, as well as the properties measured, the resulting band structures (Figure S11) show significantly dispersive bands in the trajectories in the crystallographic *c* axis (Γ → Z), while remaining relatively flat within the crystallographic *ab* plane (Γ → X → M; Z → R → A).

Due to our computational method, the *M*Pb_3_Bi structures used for the calculation are expected to be either electron-poor or electron-rich with respect to the real structure. We can examine the resulting total and partial densities of states (DOS) of the dominant atomic orbitals, shown in Figure 5, and understand adjusting the Fermi level as analogous to adjusting the experimental Pb/Bi ratio, while assuming that the general qualitative nature of the band structure remains similar. In reality, the real chemical composition and the location of the Fermi energy usually cannot be cleanly disentangled from the structure, which in turn would change the band locations and dispersions. Still, several observations can be made: in the case of M = Rh, the Fermi level clearly sits on a local maximum of the total DOS, and can be moved to a global minimum, located at an energy that is ≈ 0.7 eV higher, by addition of electrons - effectively replacing some Pb atoms by Bi. For M = Pt and Pd, the general profile of the total DOS around the Fermi energy is quite similar, as expected from them being part of the same group. Finally, when M = Au, the resulting Fermi level is located ≈ 2 eV above the Au bands, suggestive again of a clear overfilling of electrons. In addition, the large total DOS several eV below *E*_*F*_, contributed mainly by Au, is suggestive of the contribution of the aurophilic effect, potentially allowing the stabilization of AuPb_4–*x*_Bi_*x*_ with excess electrons as compared to the suggested 20 electrons per formula unit. In the future, resolving the structural disorder might allow DFT calculations of structures that are more experimentally accurate. This would allow the direct quantification and visualization of the aurophilic phenomenon, using methods such as Electron Localization Function (ELF). More broadly, this would enable the study of the underlying physics behind the empirically observed electron count, i.e., the bonding nature of the bands being filled or left empty.

**Figure 5:**
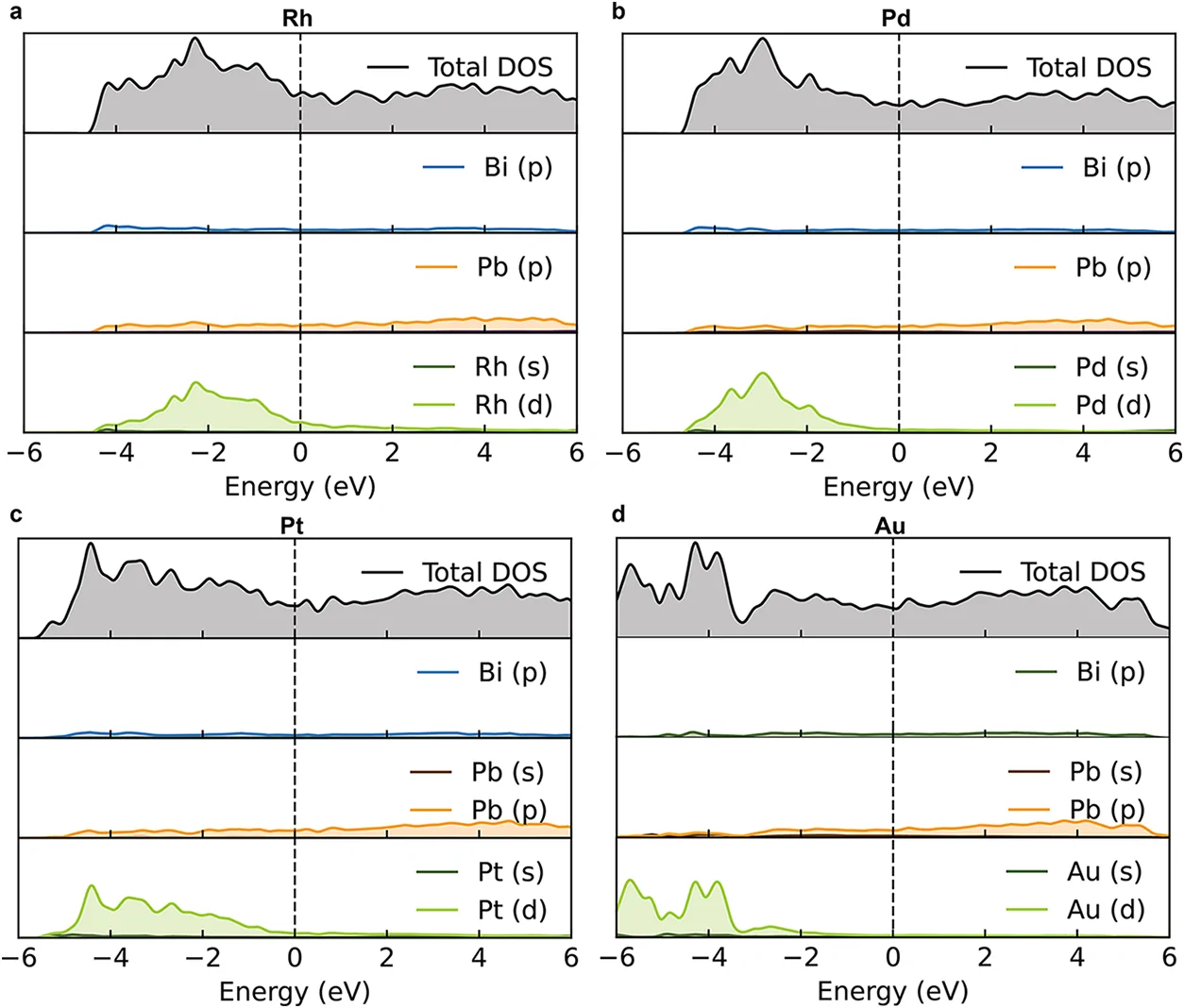
Calculated total (black lines) and partial (colored lines) density of states for *M*Pb_3_Bi structures with an idealized stoichiometry for M = Rh, Pd, Pt, and Au (a–d, respectively).

## Conclusions

In summary, we have established *M*Pb_4–*x*_Bi_*x*_ as a new family of conventional superconductors based on the PtPb_3_Bi structure type, comprising AuPb_4–*x*_Bi_*x*_, PdPb_4–*x*_Bi_*x*_, and RhPb_4–*x*_Bi_*x*_, all bulk, moderately anisotropic type-II superconductors with *T*_*c*_ between 3.4 and 4.9 K. The substitution survey defines an initial stability window in which Au, Pd, Rh, and Pt form the structure, whereas several other transition metals do not under the conditions we explored. A unifying thread is the electron count: across the series, the Pb:Bi ratio adjusts to offset the changing valence-electron count of *M*, holding the total near 20 electrons per formula unit. This points to a homogeneity range rather than a line phase, rationalizes the absence of a CdPb_4–*x*_Bi_*x*_ member (for which no composition reaches 20 electrons), and agrees with our DFT calculations, which indicate that *M* acts largely by setting the position of the Fermi level relative to features in the density of states; the same picture is mirrored in the specific heat, where the Sommerfeld coefficient tracks *T*_*c*_ across the series.

For the smaller Ni, the octagonal channels collapse into the Pb-substituted NiBi_3_ structure, and the existence of high-pressure *M*-Bi binaries such as CoBi_3_ suggests that pressure could stabilize still more members of the broader *M*–Pb-Bi family. Looking ahead, resolving the Pb and Bi site occupancies by synchrotron or neutron diffraction, extending the upper critical field measurements to higher fields, and deepening the electronic structure and bonding analysis should turn the empirical, electron-count-based stability window reported here into a predictive design rule and guide the search for higher-*T*_*c*_ members of the family.

## Supplementary Material



## Notes

EDS spectra for all compounds (Figures S1 to S4); crystallographic, atomic-position, and anisotropic displacement parameter tables (Tables S1 to S3); raw field-dependent resistivity curves (Figure S5); Meissner curves used to extract *H*_*c*1_ (Figure S6); susceptibility of NiBi_3–*x*_Pb_*x*_ (Figure S7); additional specific heat data and fits (Figures S8 to S10); and band structures calculated by DFT (Figure S11) (PDF)

## References

[1] Stewart G. R. (2015). Superconductivity
in the A15 structure. Phys. C.

[2] Chevrel R., Hirrien M., Sergent M. (1986). Superconducting
Chevrel phases: prospects
and perspectives. Polyhedron.

[3] Armitage N. P., Fournier P., Greene R. (2010). Progress and
perspectives on electron-doped
cuprates. Rev. Mod. Phys..

[4] Chu C., Deng L., Lv B. (2015). Hole-doped
cuprate high temperature
superconductors. Phys. C.

[5] Paglione J., Greene R. L. (2010). High-temperature
superconductivity in iron-based materials. Nat.
Phys..

[6] Chen X., Dai P., Feng D., Xiang T., Zhang F.-C. (2014). Iron-based high
transition temperature superconductors. Natl.
Sci. Rev..

[7] Schoop L., Hirai D., Felser C., Cava R. (2013). Superconductivity in
HfCuGe2: A non-magnetic analog of the 1111 iron pnictides. Europhys. Lett..

[8] Matthias, B. Progress in Low Temperature Physics; Elsevier, 1957; Vol. 2, pp 138–150.

[9] Gui X., Lv B., Xie W. (2021). Chemistry
in superconductors. Chem. Rev..

[10] Yao C., Ma Y. (2021). Superconducting materials:
Challenges and opportunities for large-scale
applications. Iscience.

[11] Lesser, O. ; Liu, Y. ; Maus, N. ; Panigrahi, A. ; Mallayya, K. ; Schoop, L. M. ; Gardner, J. R. ; Kim, E.-A. Learning to predict superconductivity. 2025, arXiv:2510.07373. arXiv.org e-Print archive. https://arxiv.org/abs/2510.07373.

[12] FIZ Karlsruhe ICSD: Inorganic Crystal Structure Database. 2026.

[13] Matković T., Schubert K. (1978). Kristallstruktur von
PtPb3 Bi. J. Less-Common Met..

[14] Biswas T., Schubert K. (1969). Strukturuntersuchungen
in den mischungen Pt–Tl–Pb
und Pt–Pb–Bi. J. Less-Common Met..

[15] Schubert K., Bhan S., Biswas T., Frank K., Panday P. (1968). Einige Strukturdaten
metallischer Phasen: 13. Mitteilung. Naturwissenschaften.

[16] Silva B., Luccas R., Nemes N., Hanko J., Osorio M., Kulkarni P., Mompean F., García-Hernández M., Ramos M., Vieira S., Suderow H. (2013). Superconductivity and
magnetism on flux-grown single crystals of NiBi_3_. Phys. Rev. B.

[17] Srivastava, S. ; Vardhan, Y. ; Kataria, A. ; Bawankule, P. ; Manna, P. ; Naik, P. K. ; Verma, R. ; Stewart, R. ; Lord, J. S. ; Hillier, A. D. Discovery of Quasi One Dimensional Superconductivity in PtPb3 Bi. 2026, arXiv:2604.04653. arXiv.org e-Print archive. https://arxiv.org/abs/2604.04653.

[18] Petříček V., Palatinus L., Plášil J., Dušek M. (2023). Jana2020–a
new version of the crystallographic computing system Jana. Z. Kristallogr. - Cryst. Mater..

[19] Prozorov R., Kogan V. G. (2018). Effective demagnetizing
factors of diamagnetic samples
of various shapes. Phys. Rev. Appl..

[20] Kresse G., Hafner J. (1993). Ab initio molecular
dynamics for liquid metals. Phys. Rev. B.

[21] Kresse G., Hafner J. (1994). Ab initio molecular-dynamics
simulation of the liquid-metal-amorphous-semiconductor
transition in germanium. Phys. Rev. B.

[22] Kresse G., Furthmüller J. (1996). Efficiency
of ab-initio total energy calculations for
metals and semiconductors using a plane-wave basis set. Comput. Mater. Sci..

[23] Kresse G., Furthmüller J. (1996). Efficient Iterative Schemes for Ab
Initio Total-Energy
Calculations Using a Plane-Wave Basis Set. Phys.
Rev. B.

[24] Perdew J. P., Burke K., Ernzerhof M. (1996). Generalized
Gradient Approximation
Made Simple. Phys. Rev. Lett..

[25] Blöchl P. E. (1994). Projector
augmented-wave method. Phys. Rev. B.

[26] Kresse G., Joubert D. (1999). From ultrasoft pseudopotentials
to the projector augmented-wave
method. Phys. Rev. B.

[27] Monkhorst H. J., Pack J. D. (1976). Special points for
Brillouin-zone integrations. Phys. Rev. B.

[28] M
Ganose A., Jackson A. J., Scanlon D. O. (2018). sumo: Command-line
tools for plotting and analysis of periodic *ab initio* calculations. J. Open Source Software.

[29] Gokcen N. (1992). The Bi–Pb
(bismuth-lead) system. J. Phase Equilib..

[30] Schmidbaur H., Schier A. (2008). A briefing on aurophilicity. Chem. Soc. Rev..

[31] Seibel E. M., Schoop L. M., Xie W., Gibson Q. D., Webb J. B., Fuccillo M. K., Krizan J. W., Cava R. J. (2015). Gold–Gold
Bonding: The Key to Stabilizing the 19-Electron Ternary Phases *Ln* AuSb (Ln= La–Nd and Sm). J. Am. Chem. Soc..

[32] Okamoto, H. ; Schlesinger, M. ; Mueller, E. , Eds. Alloy Phase Diagrams; ASM International, 2016.

[33] Ishida, K. ; Nishizawa, T. Bi-Co (Bismuth-Cobalt) Binary Alloy Phase Diagrams, 2nd ed.; Massalski, T. B. , Ed.; 1990; Vol. 1, pp 725–728.

[34] Leo Lukas, H. Materials Science International Team, MSIT Ag-Bi-Pb Ternary Phase Diagram Evaluation · Phase diagrams, crystallographic and thermodynamic data: Datasheet from MSI Eureka in SpringerMaterials. https://materials.springer.com/msi/literature/docs/sm_msi_r_10_012133_01. Copyright 1988 MSI Materials Science International Services GmbH.

[35] Moser Z., Dutkiewicz J., Zabdyr L., Salawa J. (1988). The Bi- Cd
(Bismuth-Cadmium)
system. Bull. Alloy Phase Diagrams.

[36] Liu Y., Zhang L., Yu D. (2009). Thermodynamic
Descriptions for the
Cd-Te, Pb-Te, Cd-Pb and Cd-Pb-Te Systems. J.
Electron. Mater..

[37] Manasijević D., Mitovski A., Minić D., Živković D., Marjanović S., Todorović R., Balanović L. (2010). Prediction
of phase equilibria and thermal analysis in the Bi–Cu–Pb
ternary system. Thermochim. Acta.

[38] Seith W., Johnen H., Wagner J. (1955). Zur Kenntnis
von Mischungslücken
im flüssigen Zustand bei metallischen Zwei-und Dreistoffsystemen. Int. J. Mater. Res..

[39] Isaeva A., Ruck M., Schäfer K., Rodewald U. C., Pöttgen R. (2015). Structure
and bonding of Bi_4_Ir: a difficult-to-access bismuth iridide
with a unique framework structure. Inorg. Chem..

[40] Schwarz U., Tencé S., Janson O., Koz C., Krellner C., Burkhardt U., Rosner H., Steglich F., Grin Y. (2013). CoBi_3_: A
Binary Cobalt-Bismuth Compound and Superconductor. Angew. Chem., Int. Ed..

[41] Tencé S., Janson O., Krellner C., Rosner H., Schwarz U., Grin Y., Steglich F. (2014). CoBi_3_–the first
binary compound of cobalt with bismuth: high-pressure synthesis and
superconductivity. J. Phys.: Condens. Matter.

[42] Badding C. K., Puggioni D., Yang J., Riesel E. A., Altman A. B., Meng Y., Fei Y., Rondinelli J. M., Freedman D. E. (2025). Predicted Ferromagnetism in Discovered
Co–Bi
Binary Phases. J. Am. Chem. Soc..

[43] Clarke S. M., Amsler M., Walsh J. P., Yu T., Wang Y., Meng Y., Jacobsen S. D., Wolverton C., Freedman D. E. (2017). Creating binary Cu–Bi compounds via high-pressure
synthesis: a combined experimental and theoretical study. Chem. Mater..

[44] Ott C., Baumgartner M., Schäfer K., Baumer F., Freitag K., Scherf L., Heletta L., Weihrich R., Pöttgen R., Nilges T. (2019). Structure and bonding of La_2_NiBi. Z. Anorg. Allg. Chem..

[45] Schäfer K., Isaeva A., Ruck M., Gerke B., Schwickert C., Poettgen R. (2014). La_2_NiSb–A Ternary Ordered Version
of the Bi3Ni Type with Highly Polar Bonding. Z. Naturforsch., B.

[46] Clogston A. M. (1962). Upper limit
for the critical field in hard superconductors. Phys. Rev. Lett..

[47] Helfand E., Werthamer N. (1966). Temperature and purity dependence
of the superconducting
critical field, *H*
_
*c*2_.
II. Phys. Rev..

[48] Clem J. R. (1998). Anisotropy
and two-dimensional behaviour in the high-temperature superconductors. Supercond. Sci. Technol..

[49] Clem J. R. (1989). Phenomenological
theory of magnetic structure in the high-temperature superconductors. Phys. C.

[50] Fujimori Y., Kan S.-i., Shinozaki B., Kawaguti T. (2000). Superconducting and
normal state properties of NiBi_3_. J. Phys. Soc. Jpn..

