# Supplementary material for: Superconductors in the PtPb3Bi Structure Type
Source: Chem Mater. 2026 Aug 19;38(17):9080–7. doi: 10.1021/acs.chemmater.6c01826 (PMC13560469; doi:10.1021/acs.chemmater.6c01826)
Supplement: Supplementary file 1 [file cm6c01826_si_001.pdf]

Supporting Information: Superconductors in the $PtPb_3Bi$ Structure Type

Lior Verbitsky, Amira Merino, Scott B. Lee, Jaime M. Moya, Sigalit Aharon, Fatmagül Katmer, Sudipta Chatterjee, Grigorii Skorupskii, Josh Leeman, Gabrielle Carrel, and Leslie M. Schoop*

Department of Chemistry, Princeton University, Princeton, New Jersey 08544, USA

E-mail: lschoop@princeton.edu

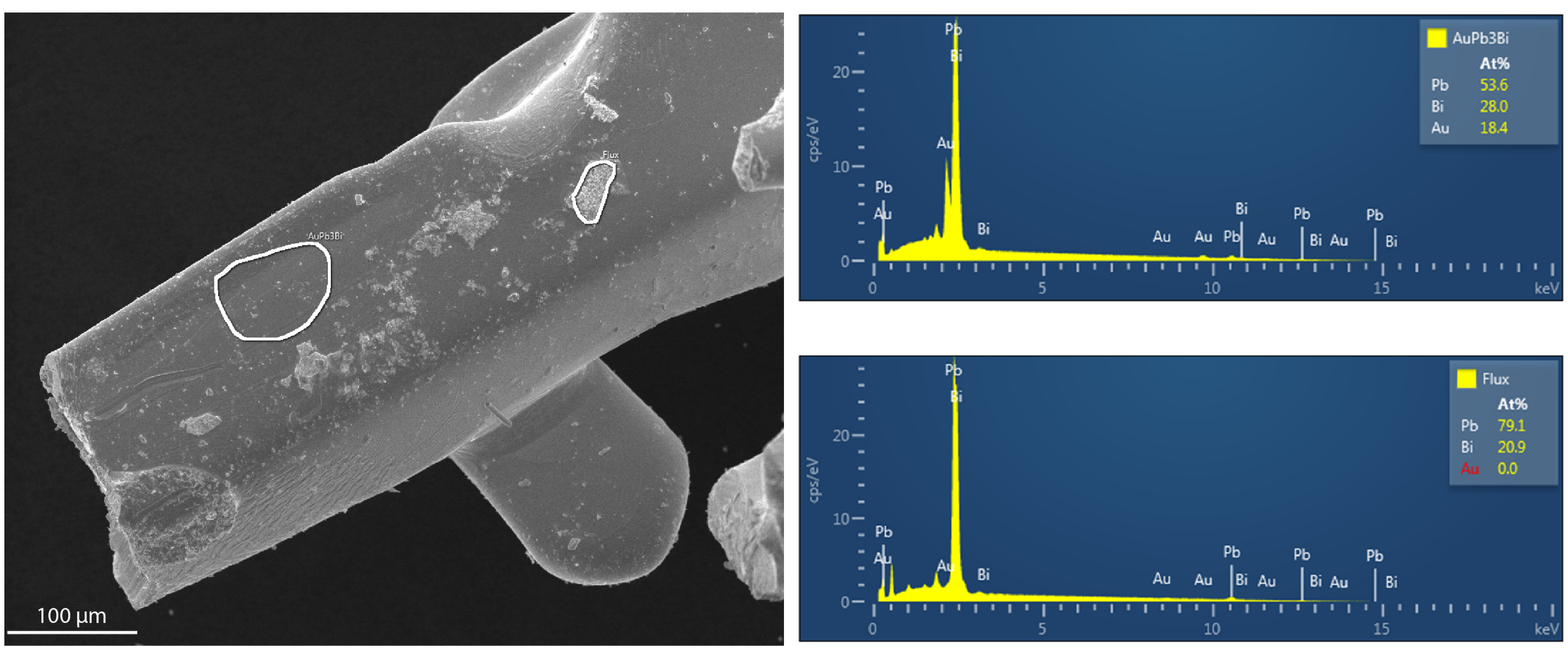

Figure S1: EDS spectra of selected clean and flux-covered regions of $AuPb_{4-x}Bi_x$, yielding the formula $AuPb_{2.9}Bi_{1.5}$.

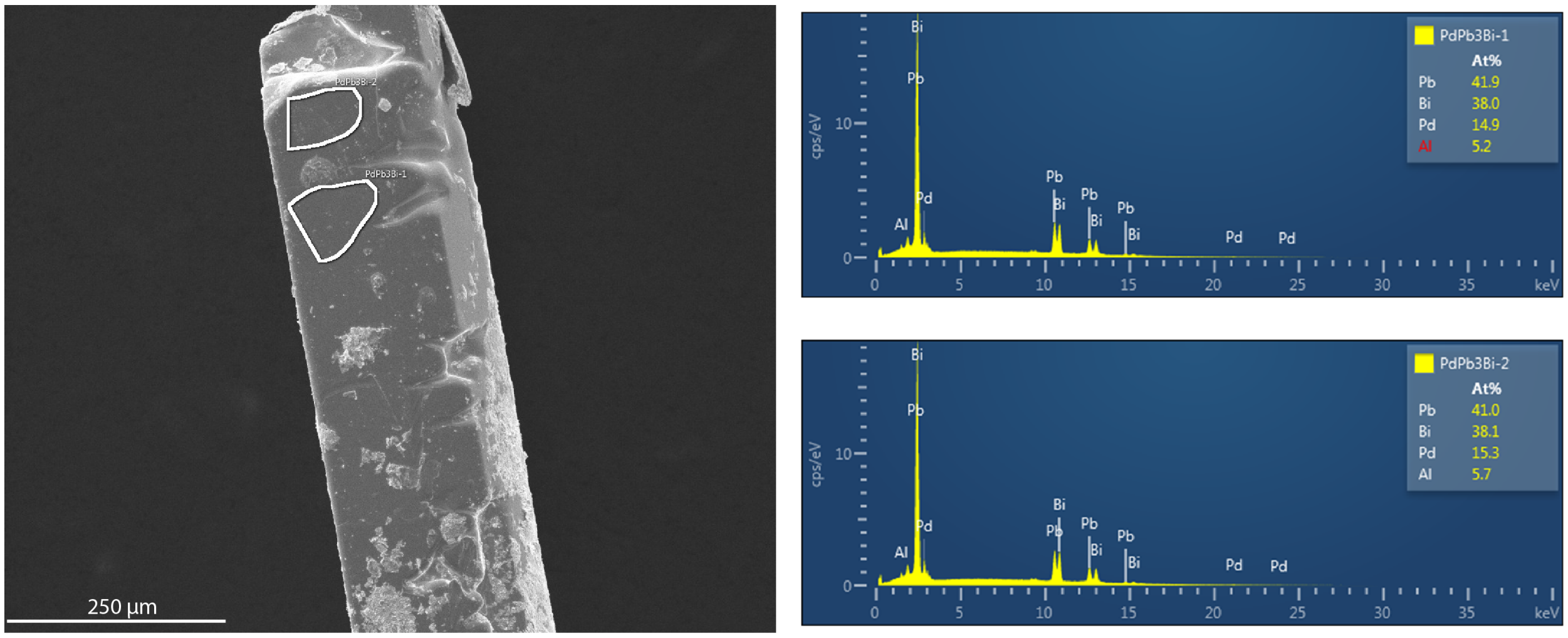

Figure S2: EDS spectra of selected clean and flux-covered regions of $PdPb_{4-x}Bi_x$, yielding the formula $PdPb_{2.8}Bi_{2.5}$. Counts from Al in the sample holder are also visible.

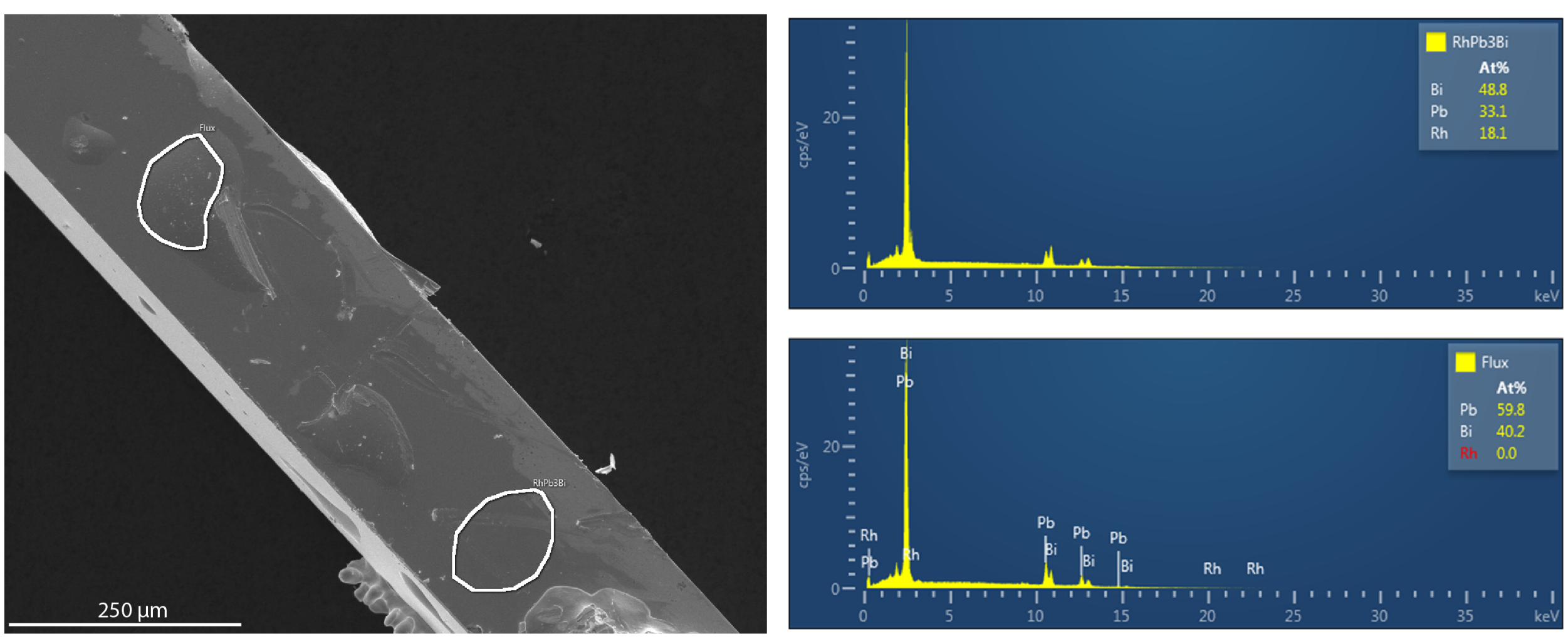

Figure S3: EDS spectra of selected clean and flux-covered regions of $RhPb_{4-x}Bi_x$, yielding the formula $RhPb_{1.8}Bi_{2.7}$.

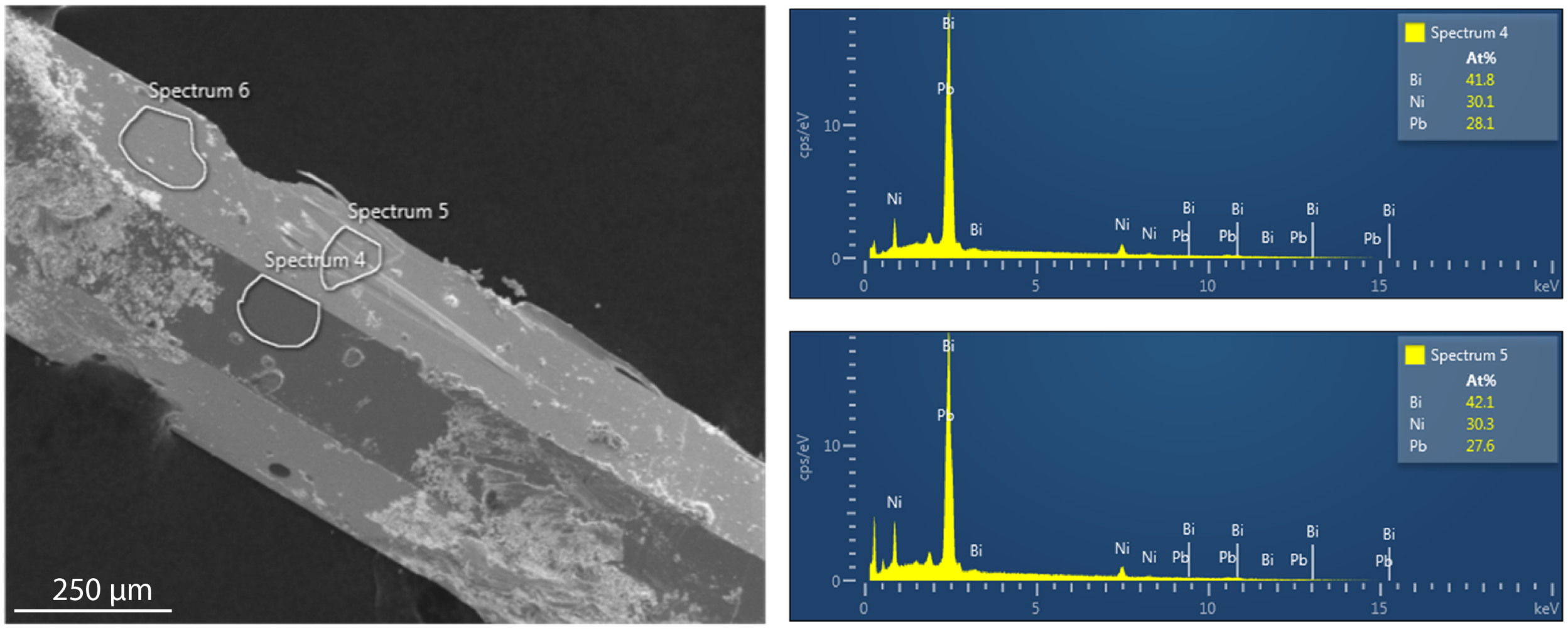

Figure S4: EDS spectra of selected clean and flux-covered regions of $NiBi_{3-x}Pb_x$, yielding the formula $NiBi_{1.4}Pb_{0.9}$.

Table S1: Crystallographic information for the reported M-Pb-Bi structures.

	Pd–Bi–Pb	Rh–Bi–Pb	Au–Bi–Pb	Ni–Bi–Pb
Crystal family	Tetragonal	Tetragonal	Tetragonal	Orthorhombic
CCDC deposit ID	2562544	2562543	2562542	2562541
Refined composition	$PdPb_2Bi_2$	$RhPb_{1.48}Bi_{2.52}$	$AuPb_{2.68}Bi_{1.32}$	$NiPb_{1.20}Bi_{1.80}$
Crystal dimensions (mm)	$0.680 \times 0.089 \times 0.031$	$0.635 \times 0.060 \times 0.048$	$0.067 \times 0.039 \times 0.027$	$0.575 \times 0.110 \times 0.067$
Radiation, λ (Å)	Mo Kα, 0.71073	Mo Kα, 0.71073	Mo Kα, 0.71073	Mo Kα, 0.71073
Absorption correction	Gaussian	Analytical	Gaussian	Gaussian
Data collection temp. (K)	293.15	293.15	293	293.15
Space group	$P4_2/mnm$	$P4_2/mnm$	$P4_2/mnm$	$Pnma$
a (Å)	11.4966(3)	11.57429(16)	11.5012	8.87104(11)
b (Å)	11.4966(3)	11.57429(16)	11.5012	4.04709(6)
c (Å)	4.09250(12)	3.95324(5)	4.297	11.47988(12)
α, β, γ (°)	90, 90, 90	90, 90, 90	90, 90, 90	90, 90, 90
Cell volume (Å^3)	540.91(2)	529.593(12)	568.3968	412.149(9)
Absorption coefficient (mm^{-1})	129.973	130.991	145.397	129.749
$\theta_{\mathrm{min}}, \theta_{\mathrm{max}}$ (°)	2.51, 30.48	2.49, 36.27	2.50, 37.48	2.90, 30.47
Refinement method	F^2	F^2	F^2	F^2
R_{int} (all, %)	19.42	14.38	11.38	12.52
Number of reflections	25247	47785	18063	16308
Number of parameters	20	20	20	26
Unique reflections ($I > 3\sigma(I)$, all)	417, 492	681, 756	561, 865	667, 708
$R(I > 3\sigma)$, $wR(I > 3\sigma)$ (%)	2.60, 6.19	3.38, 9.56	3.12, 7.89	2.88, 7.82
R(all), wR(all) (%)	3.08, 6.28	3.75, 9.71	6.40, 9.44	3.09, 7.93
$S(I > 3\sigma)$, S(all)	1.8130, 1.6885	1.9426, 1.8688	1.0410, 0.9998	1.8188, 1.7866
$\Delta\rho_{\mathrm{max}}, \Delta\rho_{\mathrm{min}}$ (e Å^{-3})	1.37, -1.36	1.68, -1.24	1.11, -1.14	2.34, -1.61

Table S2: Atomic coordinates and equivalent isotropic displacement parameters for the reported M-Pb-Bi structures.

Composition	Atom	Occ.	x	y	z	U_{eq}/U_{iso} (Å^2)
Pd–Pb–Bi	Bi1	0.5	0.29257(4)	0.70743(4)	-0.5	0.02290(15)
	Pb1	0.5	0.29257(4)	0.70743(4)	-0.5	0.02290(15)
	Bi2	0.5	0.60030(4)	0.60030(4)	0.5	0.02271(14)
	Pb2	0.5	0.60030(4)	0.60030(4)	0.5	0.02271(14)
	Pb3	0.5	0.49914(4)	0.82961(5)	0	0.02945(17)
	Bi3	0.5	0.49914(4)	0.82961(5)	0	0.02945(17)
	Pd1	1	0.41267(8)	0.58733(8)	0	0.0230(3)
Rh–Pb–Bi	Bi1	0.63	0.29303(3)	0.70697(3)	0.5	0.01679(12)
	Pb1	0.37	0.29303(3)	0.70697(3)	0.5	0.01679(12)
	Bi2	0.63	0.40290(3)	0.40290(3)	-0.5	0.01673(12)
	Pb2	0.37	0.40290(3)	0.40290(3)	-0.5	0.01673(12)
	Pb3	0.37	0.49567(3)	0.82601(4)	0	0.02371(13)
	Bi3	0.63	0.49567(3)	0.82601(4)	0	0.02371(13)
	Rh1	1	0.41278(6)	0.58722(6)	0	0.0170(2)
Au–Pb–Bi	Bi1	0.33	0.29152(4)	0.70848(4)	0	0.02975(17)
	Pb1	0.67	0.29152(4)	0.70848(4)	0	0.02975(17)
	Pb2	0.67	0.39232(4)	0.39232(4)	0	0.02838(15)
	Bi2	0.33	0.39232(4)	0.39232(4)	0	0.02838(15)
	Pb3	0.67	-0.00116(5)	0.33415(5)	0	0.03503(17)
	Bi3	0.33	-0.00116(5)	0.33415(5)	0	0.03503(17)
	Au1	1	0.08592(5)	0.08592(5)	0	0.03076(17)
Ni–Pb–Bi	Bi1	0.6	0.08654(4)	-0.25	0.68074(3)	0.01774(14)
	Pb1	0.4	0.08654(4)	-0.25	0.68074(3)	0.01774(14)
	Bi2	0.6	0.21046(4)	0.75	0.39692(3)	0.01794(14)
	Pb2	0.4	0.21046(4)	0.75	0.39692(3)	0.01794(14)
	Pb3	0.4	0.38324(5)	0.25	0.59912(3)	0.02687(16)
	Bi3	0.6	0.38324(5)	0.25	0.59912(3)	0.02687(16)
	Ni1	1	0.08745(13)	0.25	0.52078(11)	0.0150(3)

Table S3: Anisotropic displacement parameters for the reported M-Pb-Bi structures. All values are given in Å^2.

Composition	Atom	U_{11}	U_{22}	U_{33}	U_{12}	U_{13}	U_{23}
Pd–Pb–Bi	Bi1	0.0232(2)	0.0232(2)	0.0224(3)	0.0027(2)	0	0
	Pb1	0.0232(2)	0.0232(2)	0.0224(3)	0.0027(2)	0	0
	Bi2	0.0236(2)	0.0236(2)	0.0210(3)	-0.0012(2)	0	0
	Pb2	0.0236(2)	0.0236(2)	0.0210(3)	-0.0012(2)	0	0
	Pb3	0.0280(3)	0.0250(3)	0.0353(3)	-0.00138(16)	0	0
	Bi3	0.0280(3)	0.0250(3)	0.0353(3)	-0.00138(16)	0	0
	Pd1	0.0231(4)	0.0231(4)	0.0228(5)	0.0049(4)	0	0
Rh–Pb–Bi	Bi1	0.01627(18)	0.01627(18)	0.0178(3)	0.00226(14)	0	0
	Pb1	0.01627(18)	0.01627(18)	0.0178(3)	0.00226(14)	0	0
	Bi2	0.01649(17)	0.01649(17)	0.0172(3)	-0.00178(14)	0	0
	Pb2	0.01649(17)	0.01649(17)	0.0172(3)	-0.00178(14)	0	0
	Pb3	0.0225(2)	0.0185(2)	0.0301(3)	-0.00125(12)	0	0
	Bi3	0.0225(2)	0.0185(2)	0.0301(3)	-0.00125(12)	0	0
	Rh1	0.0170(3)	0.0170(3)	0.0170(5)	0.0037(3)	0	0
Au–Pb–Bi	Bi1	0.0275(2)	0.0275(2)	0.0343(4)	0.0029(3)	0	0
	Pb1	0.0275(2)	0.0275(2)	0.0343(4)	0.0029(3)	0	0
	Pb2	0.0261(2)	0.0261(2)	0.0328(3)	-0.0028(2)	0	0
	Bi2	0.0261(2)	0.0261(2)	0.0328(3)	-0.0028(2)	0	0
	Pb3	0.0330(3)	0.0293(3)	0.0428(3)	0.00162(19)	0	0
	Bi3	0.0330(3)	0.0293(3)	0.0428(3)	0.00162(19)	0	0
	Au1	0.0287(2)	0.0287(2)	0.0350(4)	-0.0052(3)	0	0
Ni–Pb–Bi	Bi1	0.0220(3)	0.0160(3)	0.0152(2)	0	-0.00298(11)	0
	Pb1	0.0220(3)	0.0160(3)	0.0152(2)	0	-0.00298(11)	0
	Bi2	0.0181(3)	0.0165(3)	0.0192(2)	0	0.00505(11)	0
	Pb2	0.0181(3)	0.0165(3)	0.0192(2)	0	0.00505(11)	0
	Pb3	0.0162(2)	0.0405(3)	0.0239(3)	0	-0.00402(13)	0
	Bi3	0.0162(2)	0.0405(3)	0.0239(3)	0	-0.00402(13)	0
	Ni1	0.0151(6)	0.0130(6)	0.0169(6)	0	-0.0011(4)	0

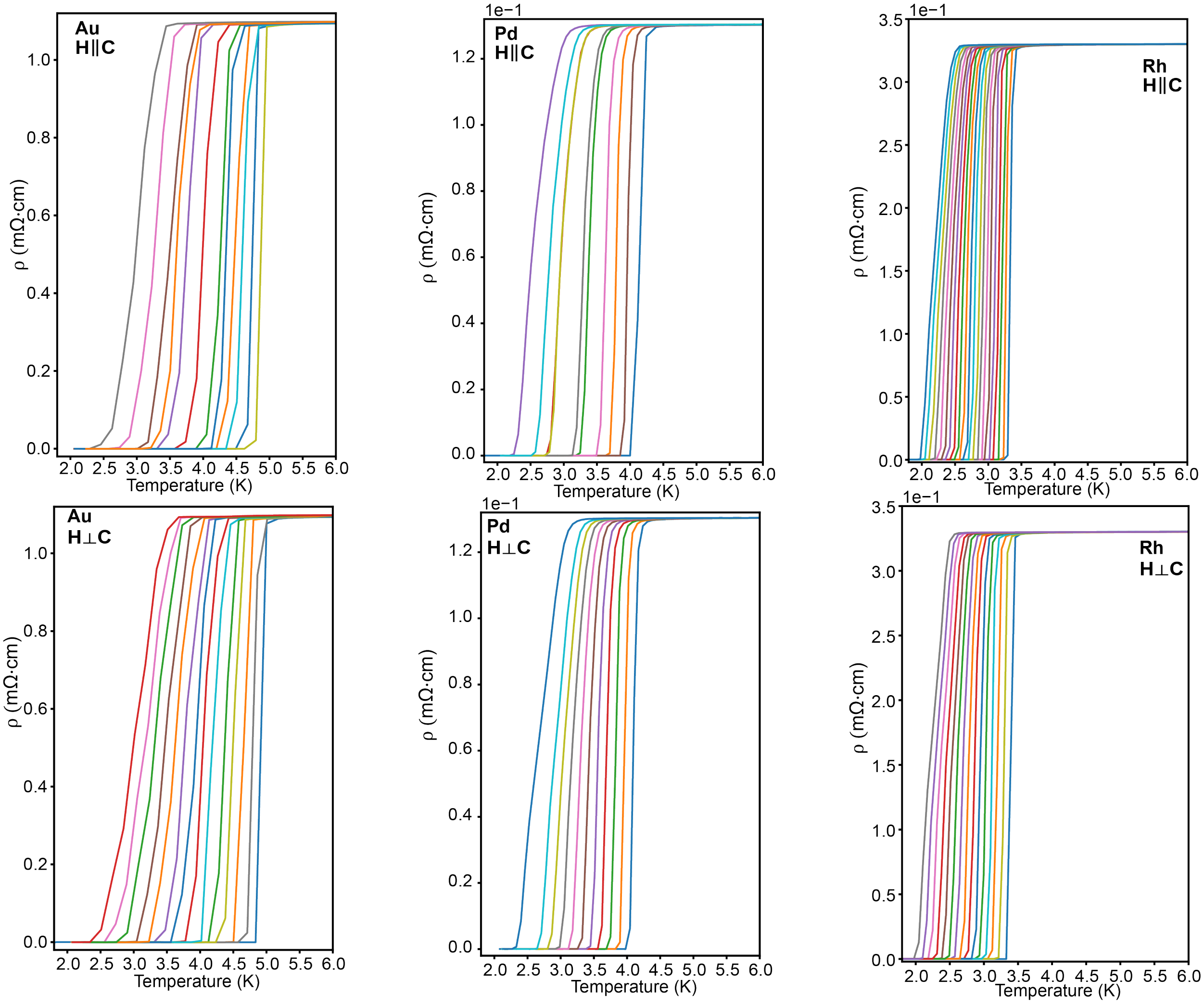

Figure S5: Raw resistivity under increasing magnetic field, parallel and perpendicular to the crystallographic c axis.

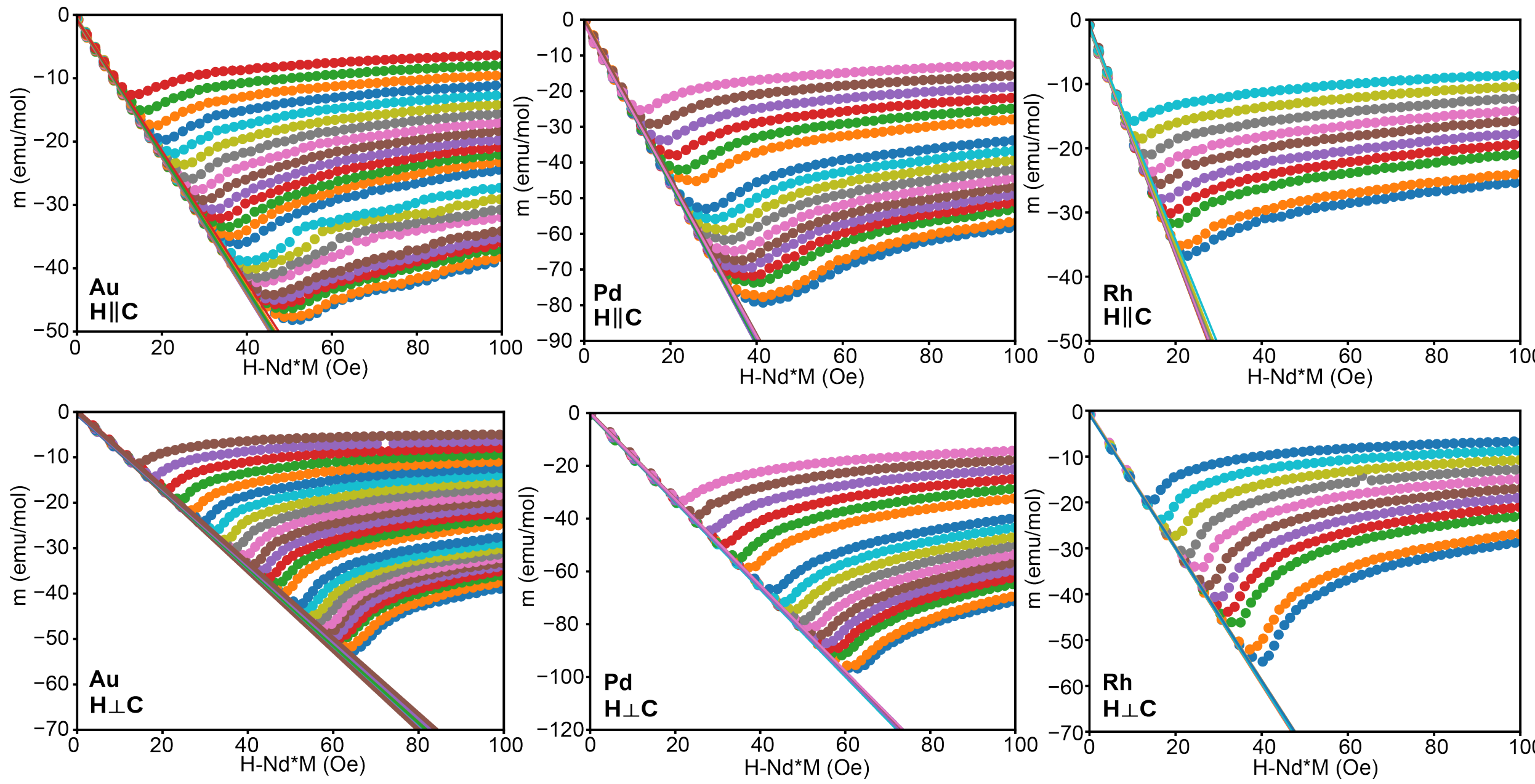

Figure S6: Raw magnetic moment versus magnetic field with increasing temperature. Lines are linear fits to the Meissner curve.

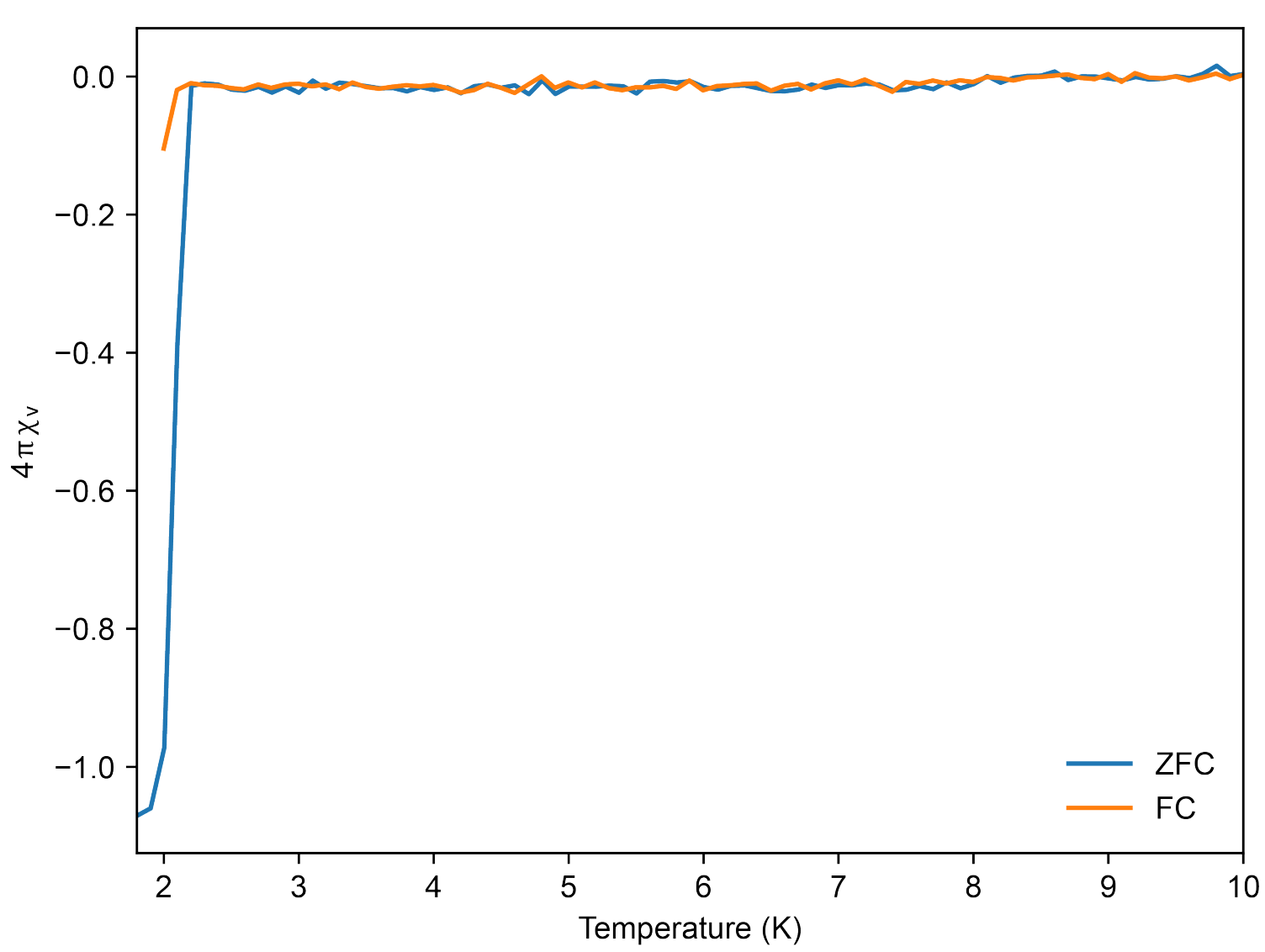

Figure S7: Demagnetization-corrected ZFC and FC magnetic susceptibility of $NiBi_{3-x}Pb_x$ under a 2 Oe bias field.

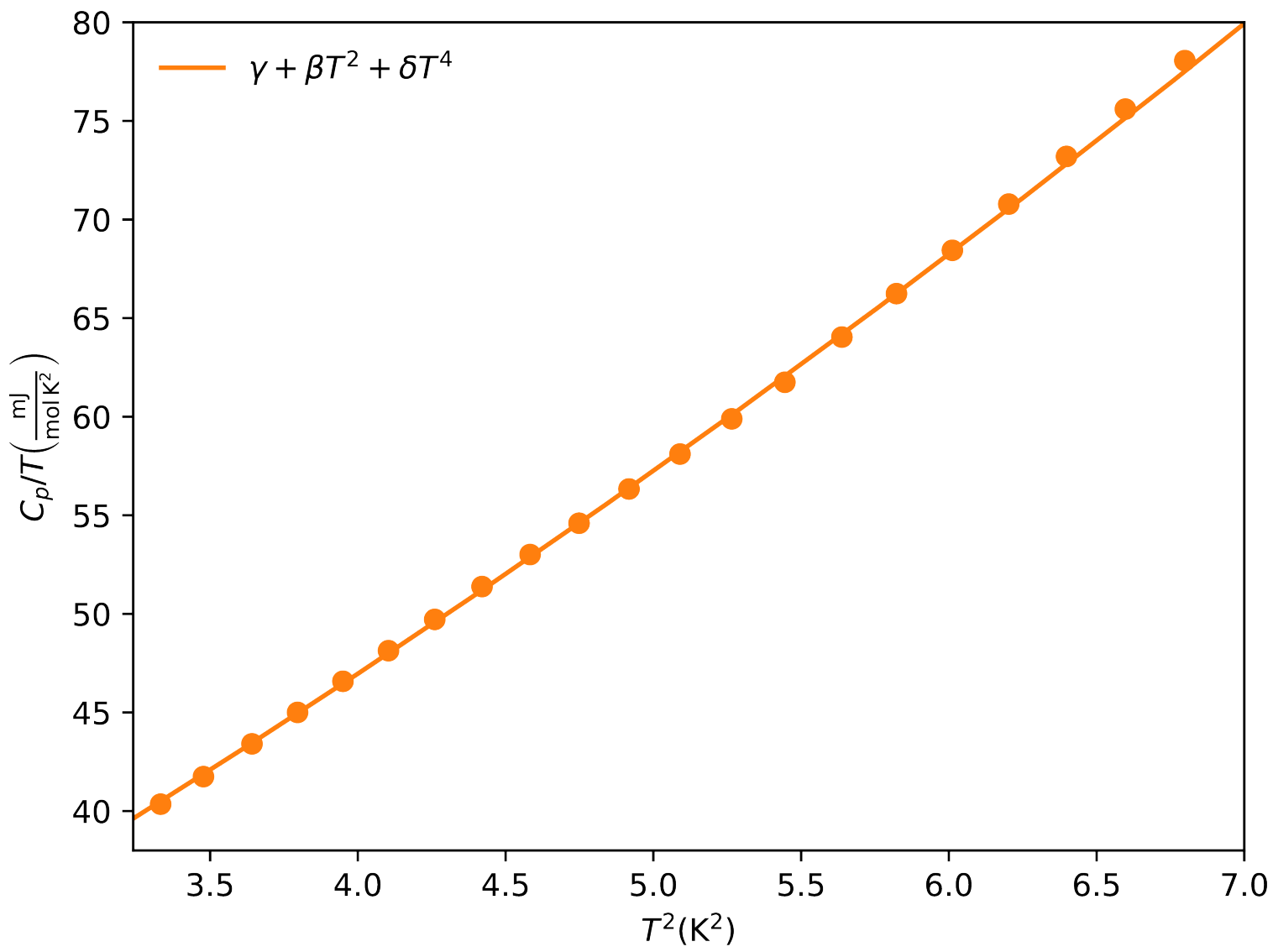

Figure S8: Low-temperature measurement and fit of the total specific heat C_p/T for $AuPb_{4-x}Bi_x$ in a 50 kOe field parallel to the crystallographic ab plane.

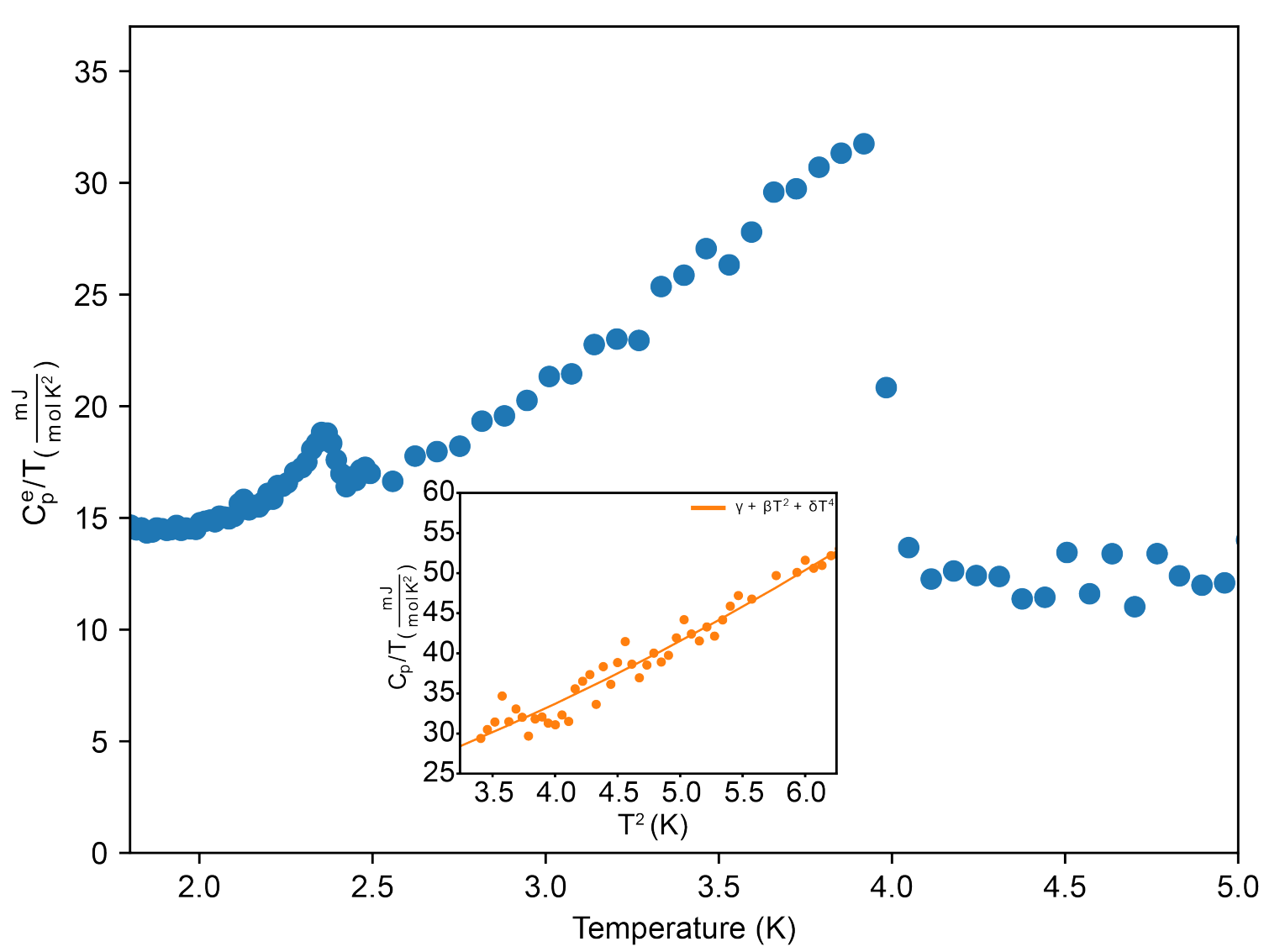

Figure S9: Electronic specific heat C_p^e/T of $PdPb_{4-x}Bi_x$ in zero field. An unidentified transition is observed also at ≈ 2.4 K. Inset: low-temperature measurement and fit of the total specific heat C_p/T in a 50 kOe field parallel to the crystallographic ab plane.

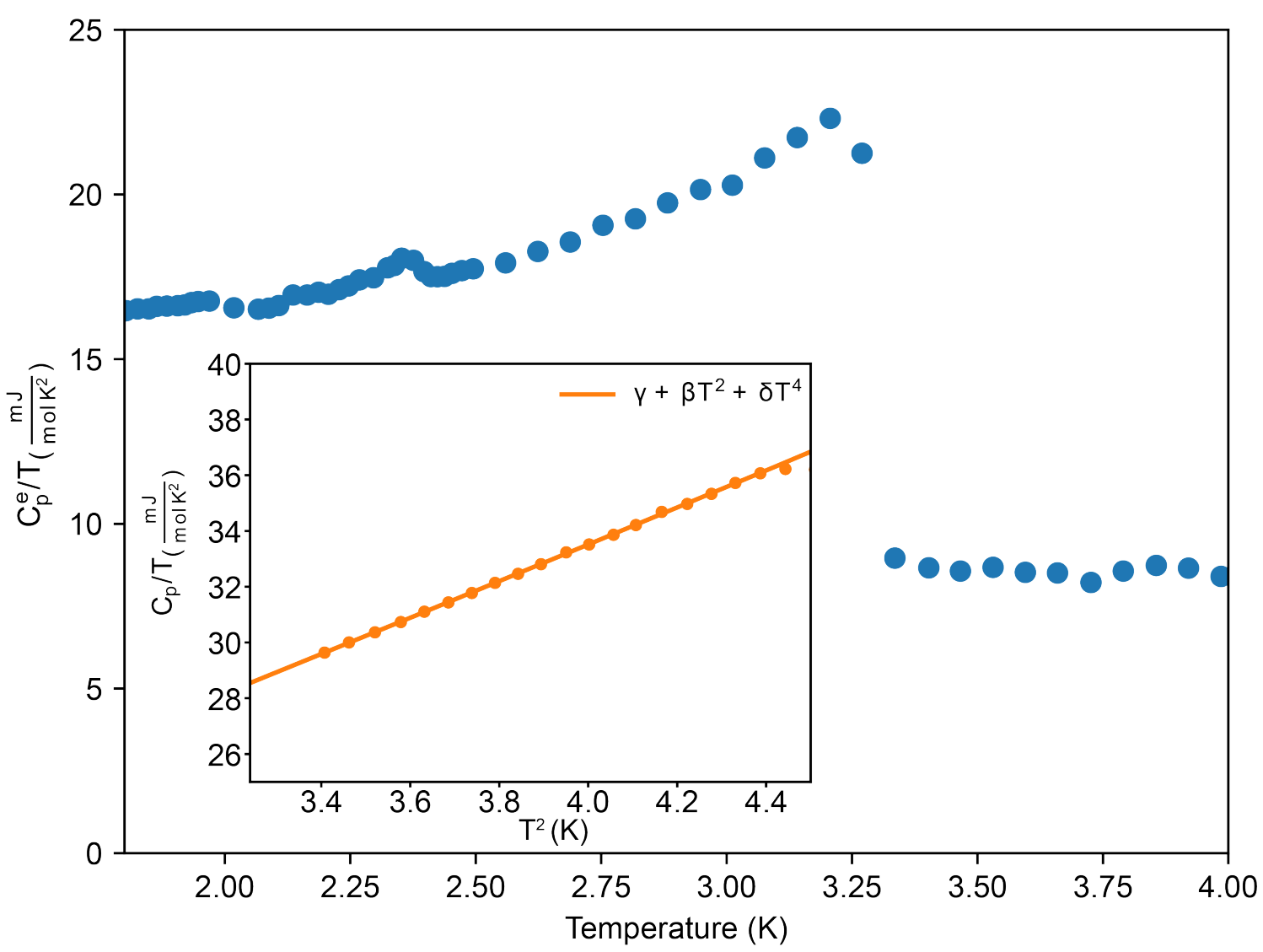

Figure S10: Electronic specific heat C_p^e/T of $RhPb_{4-x}Bi_x$ in zero field. An unidentified transition is observed also at ≈ 2.4 K. Inset: low-temperature measurement and fit of the total specific heat C_p/T in a 50 kOe field parallel to the crystallographic *ab* plane.

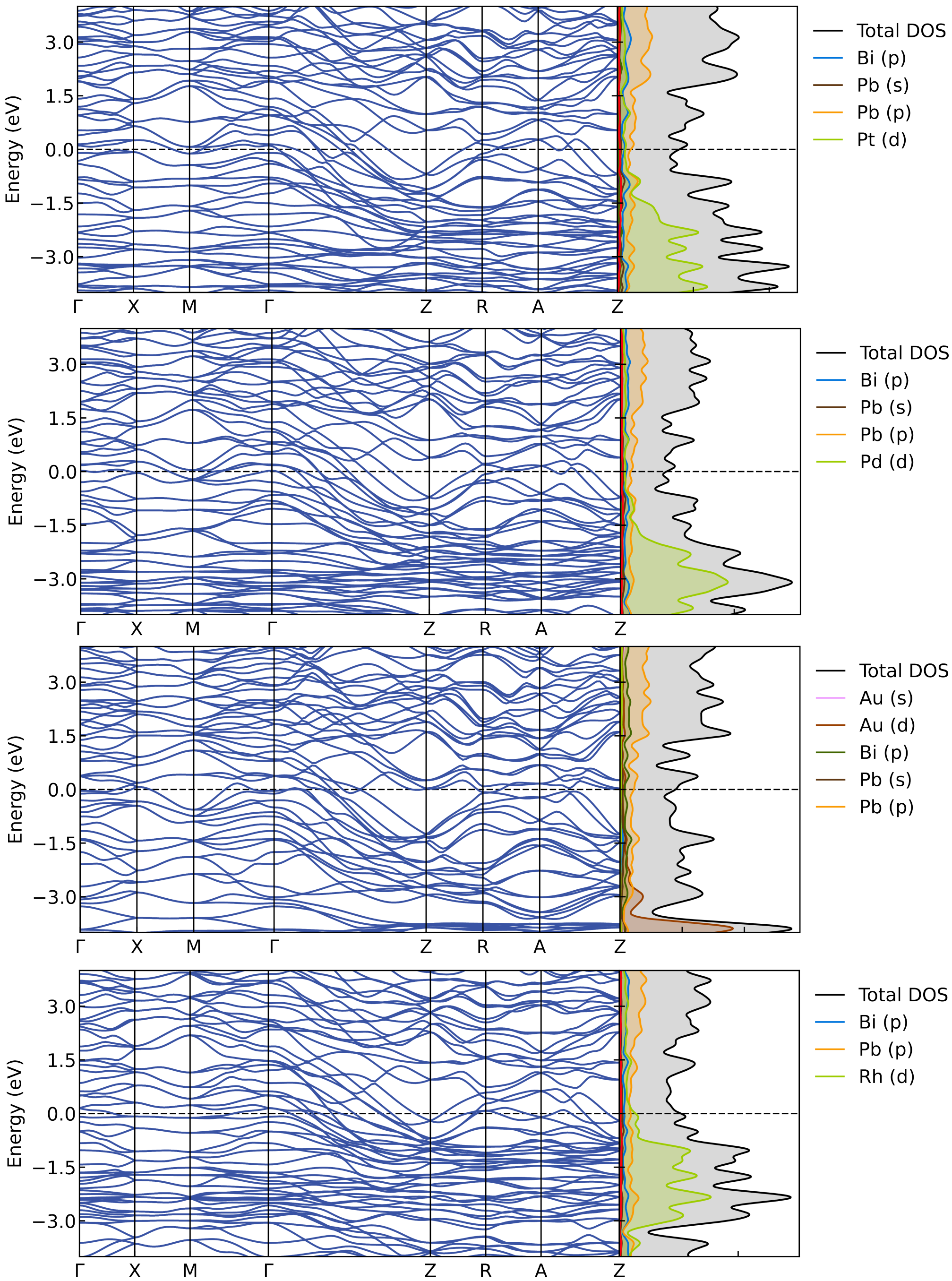

Figure S11: Calculated band structures and total and partial density of states for the $M\mathrm{Pb}_{4-x}\mathrm{Bi}_x$ structures.
